# Pioglitazone Treatment Reduces Adipose Tissue Inflammation through Reduction of Mast Cell and Macrophage Number and by Improving Vascularity

**DOI:** 10.1371/journal.pone.0102190

**Published:** 2014-07-10

**Authors:** Michael Spencer, Lin Yang, Akosua Adu, Brian S. Finlin, Beibei Zhu, Lindsey R. Shipp, Neda Rasouli, Charlotte A. Peterson, Philip A. Kern

**Affiliations:** 1 The Department of Medicine, Division of Endocrinology, and the Barnstable Brown Diabetes and Obesity Center, University of Kentucky, Lexington, Kentucky, United States of America; 2 The College of Public Health, University of Kentucky, Lexington, Kentucky, United States of America; 3 The College of Health Sciences, University of Kentucky, Lexington, Kentucky, United States of America; 4 The University of Colorado Denver, Division of Endocrinology, and Eastern Colorado Veterans Health Care System, Denver, Colorado, United States of America; Virginia Tech, United States of America

## Abstract

**Context and Objective:**

Adipose tissue in insulin resistant subjects contains inflammatory cells and extracellular matrix components. This study examined adipose pathology of insulin resistant subjects who were treated with pioglitazone or fish oil.

**Design, Setting and Participants:**

Adipose biopsies were examined from nine insulin resistant subjects before/after treatment with pioglitazone 45 mg/day for 12 weeks and also from 19 subjects who were treated with fish oil (1,860 mg EPA, 1,500 mg DHA daily). These studies were performed in a clinical research center setting.

**Results:**

Pioglitazone treatment increased the cross-sectional area of adipocytes by 18% (p = 0.01), and also increased capillary density without affecting larger vessels. Pioglitazone treatment decreased total adipose macrophage number by 26%, with a 56% decrease in M1 macrophages and an increase in M2 macrophages. Mast cells were more abundant in obese versus lean subjects, and were decreased from 24 to 13 cells/mm^2^ (p = 0.02) in patients treated with pioglitazone, but not in subjects treated with FO. Although there were no changes in total collagen protein, pioglitazone increased the amount of elastin protein in adipose by 6-fold.

**Conclusion:**

The PPARγ agonist pioglitazone increased adipocyte size yet improved other features of adipose, increasing capillary number and reducing mast cells and inflammatory macrophages. The increase in elastin may better permit adipocyte expansion without triggering cell necrosis and an inflammatory reaction.

## Introduction

Insulin resistance develops with obesity and in the setting of inadequate β-cell response, leads to type 2 diabetes (T2DM). However, this process is complex, and many changes occur in the adipose tissue of insulin resistant subjects, including the development of a proinflammatory environment, characterized by an influx of macrophages, an alteration in collagen and other extracellular matrix (ECM) components, and hypoxia due to decreased capillaries [1]–[4]. A leading hypothesis behind these changes in adipose tissue is that increased adipocyte size in the setting of a relatively rigid ECM leads to a compromised blood supply, adipocyte necrosis, inflammation, and fibrosis [5].

A number of treatments have been used to decrease adipose tissue inflammation and reduce insulin resistance. The PPARγ-agonist thiazolidinedione (TZD) drugs are used to treat T2DM. TZDs improve peripheral insulin sensitivity, and have a spectrum of anti-inflammatory properties, including a reduction in plasma inflammatory markers, and a reduction in adipose tissue macrophages [6]–[9]. Fish oils contain ω-3 polyunsaturated fatty acids (ω-3 PUFA), which also have anti-inflammatory properties [10], [11] and have recently been shown to reduce plasma MCP-1 and adipose macrophages [12]. In some settings, ω-3 PUFA consumption resulted in an increase in adiponectin through a PPARγ-dependent mechanism [13], [14].

In addition to macrophages, adipose tissue contains other inflammatory cells, and several recent studies have identified mast cells in adipose tissue of both mice and humans [15], [16]. When adipose mast cells were reduced through genetic or drug manipulation, the metabolic profiles of mice were improved [16]. In another study of humans, mast cell number was no different between obese and lean humans, however the degree of mast cell activation was higher with obesity, and the overall number of mast cells was higher in patients with T2DM [17].

In this study, we examined in greater depth the response of adipose tissue macrophages to pioglitazone and fish oil treatment, and to determine whether anti-inflammatory treatments would reduce adipose mast cells. Pioglitazone, but not fish oil, treatment resulted in a decrease in adipose tissue mast cells. In addition, pioglitazone increased adipocyte size, increased adipose tissue capillaries, and increased elastin in adipose tissue.

## Materials and Methods

### Ethics Statement

All research methods pertaining to human subjects were approved by Institutional Review Boards from either University of Arkansas for Medical Sciences or the University of Kentucky. This included obtaining consent from all participants using consent forms that were approved by the above named Institutional Review Boards.

### Human Subjects

Three groups of participants were analyzed for this study to determine the effects of obesity, pioglitazone treatment, and fish oil treatment, as outlined in Table 1. To study the effects of obesity, a group of subjects were recruited that included 7 lean, normal glucose tolerant subjects, who were compared with 9 obese subjects. For the study of pioglitazone, we analyzed the adipose tissues of 9 subjects with obesity and impaired glucose tolerance who had been treated with pioglitazone. These subjects were part of a study on the effects of pioglitazone on adipose inflammation and reported previously [6], [7]. All of these subjects had impaired glucose tolerance on a 75 g oral glucose tolerance test, and were on no medications that could affect carbohydrate or lipid metabolism. After a two week dose escalation, they were treated with pioglitazone 45 mg per day for 10 weeks, which yielded a 65% increase in insulin sensitivity [18], and adipose biopsies were performed by incision from the lower abdominal wall. In the nine subjects treated with pioglitazone in this study, S_I_ increased from 1.6 to 2.3 units (p<0.01), and 2 hr glucose decreased from 161 to 127 (p<0.05), and BMI increased from 32 to 33 (p<0.05), all consistent with previous reports [18], [19]. The third group of subjects were obese, had at least 3 features of metabolic syndrome (14 out of 19 had IGT), and all subjects had fat biopsies before and after 12 weeks of treatment with fish oil (omega-3-Acid Ethyl Esters, Lovaza, 4 g/day) for 12 weeks. As reported previously [12], fish oil treatment did not affect insulin sensitivity or glucose tolerance, and reduced triglycerides by 20% (p<0.05). All participants signed consent forms that were approved by the Institutional Review Boards from either the University of Arkansas for Medical Sciences or the University of Kentucky. No participants had any history of coronary disease, inflammatory disease, the chronic use of any anti-inflammatory medication or other medication likely to change adipocyte metabolism; none were taking ω-3 fatty acids supplements, and routine baseline labs (liver function, creatinine, TSH, CBC) were normal.

**Table 1:** Baseline characteristics of study subjects.

	Lean	Obese	Pioglitazone-treated	Fish oil-treated
Number/female	7/5	9/8	9/8	19/13
Age (yr)	40±2.8	46.56±3.16	50±2.6	48.8±2.3
% body fat	27.95±3.5	45.19±1.0	41±2.4	44.4±2.7
BMI (kg/m^2^)	23.8±0.93	35.9±0.81	32±1.5	33.4±2.3
Triglycerides (mg/dl)	70.6±9.14	140.1±13.89	233±48.5	147.3±7.7
Cholesterol (mg/dl)	190±9.25	159.7±12.89	218±20.25	207.1±6.4
HDL (mg/dl)	62±7.84	49.0±3.86	48±2.3	53.5±4.2
LDL (mg/dl)	113.75±9.1	82.4±11.64	117±18.1	125.9±6.1
Glucose fasting (mg/dl)	85.25±3.59	92.2±3.51	90±4.5	95.8±2.8
Glucose 2 hr (mg/dl)	121.9±14.14	159.9±7.49	161±8.7	167.8±6.6
HbA1c (%)	5.3±0.13	5.8±0.18	5.4±0.13	6.0±0.6
S_I_ (×10−4 min−1 [µU/ml]−1)	7.02±1.39	1.8±0.22	1.6±0.16	1.9±0.9

Participants in this study were recruited from previously reported clinical trials registered at clinicaltrials.gov, and a number of publications have already come from these studies. NCT00108615 (recruitment from 2004 through 2007) involved participants treated with pioglitazone and different outcomes from this study have been described previously [7], [18], [20]–[23]. NCT00470262 (recruitment from 2007 through 2012) also involved participants treated with pioglitazone [24]. NCT00579436 (recruitment 2007 through 2013) involved the treatment of subjects with fish oils or placebo [12]. The examination of adipose mast cells, or the other features of adipose tissue reported herein, were not part of the original design of these studies.

Insulin sensitivity was measured with a frequently sampled IV glucose tolerance test (FSIGT), which yields a measure of insulin sensitivity (S_I_) that correlates well with glucose disposal rate from the euglycemic clamp [4], [25], [26].

#### Histochemistry and immunohistochemistry

Adipose samples were fixed in Bouin’s, paraffin embedded and cut into 5 µm thick sections. All macrophages were stained with CD68 (Dako, clone KP1,) followed by HRP-linked secondary antibody and DAB substrate for color development (ImmPACT DAB, Vector Labs, SK-4105). Identification of macrophage polarization used a triple-staining procedure which used CD68, CD86 (Santa Cruz Biotech, SC-28347, to identify M1 macrophages) and CD206 (R and D Systems, AF2534, to identify M2 macrophages), as described previously [1]. HRP-linked secondary antibodies and three different chromogenic substrates were used to localize the primary antibodies: ImmPACT NovaRED (Vector Labs, SK-4805), ImmPACT VIP (Vector Labs, SK-4605), and TrueBlue (KPL, 50-78-02). The Nuance multispectral microscopy camera (PerkinElmer, 130925) was used to separate the chromogenic substrates and identify cells stained with the different antibodies and determine any co-localization.

The quantitation of blood vessels was performed by double staining for endothelial cells (UEA lectin, Sigma: L8262) and alpha smooth muscle actin (ASMA, Santa Cruz Biotech: SC130616), as describe previously [4]. HRP-secondary antibody and ImmPACT SG (Vector Labs, SK-4705) chromogenic substrate was used to develop the staining for ASMA. Endothelial cells stained with UEA were developed with streptavidin-HRP conjugate (Vector Labs, SA-5004) followed by chromogenic substrate ImmPACT NovaRED. The Nuance multispectral microscopy system was used to separate the chromogenic substrates. Capillaries stained only with the endothelial stain, whereas larger blood vessels were identified by the ASMA ring around the endothelial cells [4].

Adipose mast cells were identified by staining for mast cell tryptase (Santa Cruz Biotechnology, sc-33676) followed by chromogenic color development using ImmPACT VIP.

### Quantification of images

Single stained images (CD68 alone and Mast Cells) were captured on Nikon 55i upright microscope using Nikon NIS Elements software. Images were quantified in the NIS Elements application by manual counting the number of cells. The total area of adipose tissue was measured for each image and used to normalize results as cells per mm^2^.

Multiple stained images (macrophage polarization and vessels) were imaged on the Nuance system and individual spectral profiles for each chromogen were used to separate the different antigens and to create a multichannel image. This creates a image channel for each chromogen. The different images were manually counted with the number of cells being expressed as cells/mm^2^ of adipose tissue.

### Measurement of adipocyte size

**Figure 1:**
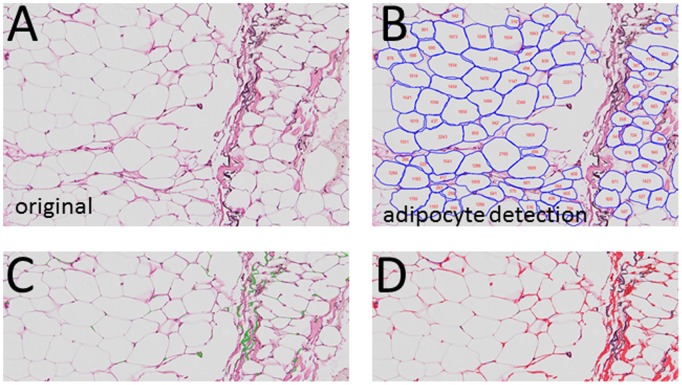
Adipose immunohistochemistry for cell size and fibrosis. Adipose tissue was stained for elastin, as described in Methods. A. Elastin stain, illustrating the outline of the adipocytes, elastin (black stain) and areas of fibrosis (pink). B. Identification of adipocytes using the image analysis software, and the assignment of adipocyte area to the cells. C. Enhancement of image to bring out elastin (green). D. Enhancement of image to bring out collagens (red).

Adipocyte size, elastin, and collagen were identified from adipose tissue stained with Accustain Elastic Stain (Sigma, HT25A-1KT). This technique stains elastin fibers black and collagen pink, and adipocytes are easily identified as unstained areas surrounded by collagen (Figure 1).

To measure adipocyte size, these images were analyzed by a machine learning-based algorithm for quantification of adipocyte cross-sectional area analysis (CSA). The image segmentation algorithm have two major steps: 1) Machine learning based automated detection of the geometric center of the adipocytes, 2) a gradient vector flow based deformable model that adopts color gradients to define the adipocyte boundaries and ECM. A detailed description of the automated image segmentation algorithm can be found in Methods S1. The accuracy of the automated algorithm for adipocyte detection was determined by comparing results to manually measured adipocyte areas. In a previous study on the effects of fish oil on adipose inflammation [12], we measured adipocyte size manually by sizing 300–600 cells in the sections. To compare this method to the automated algorithm, we compared the manually derived cell sizing to the automated method. As shown in Figure S1, mean adipocyte size was highly correlated between the two methods (r = 0.81, p<0.01). The automated method has the advantage of high throughput analysis of all the adipocytes on the section, between 8000–12000 cells, which is more than the number of cells that can be practically measured manually. The automated method improves accuracy, avoids the problem of measuring cells in non-representative fields, and also saves tremendous amount of time and labor. A similar automated approach was recently validated for quantification of muscle fiber CSA [27].

### Quantitation of collagen and elastin

As described above, the Accustain identified collagen and elastin based on their pink and black staining, respectively. To quantify the amount of collagen and elastin in the tissue section, the same techniques were used as described above, and explained in details in Methods S1, but color thresholding of the ECM segmentation was calculated based on the automated segmentation algorithm to calculate the percent collagen and elastin [28], [29].

### Statistical Analysis

All data from samples were expressed as mean ± SEM. Statistical analysis was performed in either Microsoft Excel or GraphPad Prism applications. Parametric t-tests were used to detect significance between pre/post data or lean/obese data. Macrophage polarization was tested using both ANOVA with Tukey post-tests and t-test between different groups. ANOVA and t-test produced identical results in terms of which groups were significant. Histograms of adipocyte cross-sectional area were analyzed in MATLAB with final graphs being produced in Microsoft Excel.

## Results

During the development of obesity-mediated insulin resistance, adipocyte size increases, followed by varying degrees of fibrosis, and inflammation [5]. Pioglitazone has well-described anti-inflammatory properties, yet the induction of PPARγ would be expected to increase adipogenesis, lipogenesis, and increase fat cell size. Previous studies have examined adipocyte size in response to TZDs, but with varying results [30]–[34]. To examine the effects of pioglitazone on adipose cellularity, the adipose tissue from 9 subjects was analyzed histochemically pre- and post-piloglitazone, and image analysis software was used to determine adipocyte size in cross section. A variety of methods can be used to stain adipose tissue, but we used the stain for elastin, as described in methods, since the adipocytes were well outlined, and this method allowed the simultaneous staining for collagens and elastin. Representative images are shown in Figure 1, where Figure 1A is adipose stained for elastin, and Figure 1B shows the identification of adipocytes through the image analysis software. Figures 1C and 1D are pseudo-colored to bring out the staining for elastin (green in Figure 1C) and collagen (red in Figure 1D).

**Figure 2:**
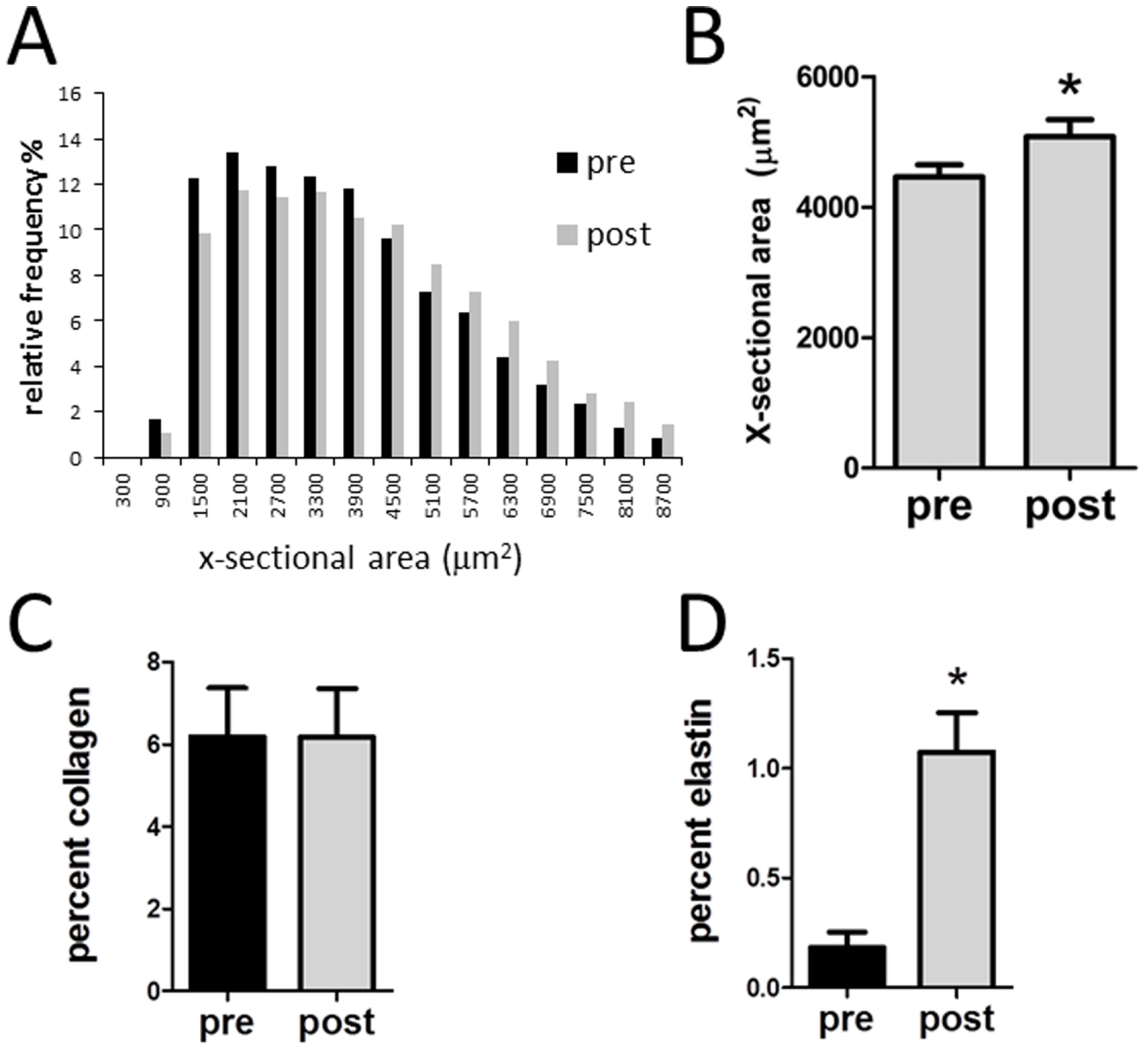
Quantitation of images pre and post pioglitazone. A. Cell size from Figure 1B was measured in all adipocytes from a whole slide scan, representing 8000–12000 cells, and the distribution of cell cross-sectional area relative frequency is shown before and after pioglitazone treatment. B. Average adipocytes cross-sectional area. C. Analysis of images illustrated in Figure 1D for overall collagen. D. Analysis of images illustrated in Figure 1C for elastin. *p<0.05 vs pre-pioglitazone.

All the adipocytes from each histological section were identified and cross sectional area (CSA) determined (8,000–12,000 cells per slide). As shown in Figure 2A, subjects demonstrated wide variety of fat cell size and contained a distribution of cells varying from 500 to 8700 µm^2^. There was an overall decrease in the frequency of small adipocytes and an increase in the frequency of larger adipocyte following pioglitazone treatment (Figure 2A), such that the average fat cell size was significantly increased following pioglitazone treatment (Figure 2B).

The images in Figure 1 were then analyzed for ECM components and these data were expressed in terms of the space occupied by collagen and elastin. As shown in Figure 2C, there was no change in overall collagen content in adipose tissue following pioglitazone. However, pioglitazone treatment resulted in a significant increase in adipose elastin (Figure 2D).

**Figure 3:**
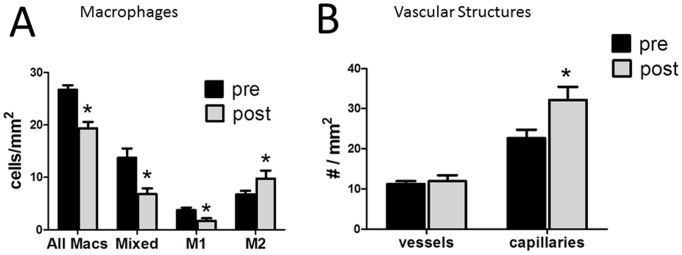
Adipose macrophage polarity and vascularity following pioglitazone treatment. A. Macrophages were characterized as M1, M2, or mixed, as described in Methods, pre and post pioglitazone treatment. B. Adipose blood vessels were characterized as capillaries, or vessels, based on the absence/presence of ASMA staining of a vessel wall (see Methods) and counted in adipose sections pre and post pioglitazone. *p<0.05 vs pre pioglitazone.

Previous studies have demonstrated that treatment of insulin resistant subjects with TZDs resulted in less inflammation, including a decrease in adipose tissue macrophage number and cytokine expression [6], [35], but no previous study has examined the effects of TZDs on macrophage polarity. Therefore, we examined adipose tissue macrophages immunohistochemically; CD68 positive cells were co-stained for the M1 and M2 markers CD86 and CD206, respectively. As described previously [1], human adipose tissue contained many macrophages that are of a mixed phenotype, staining with both the M1 and M2 markers, and most macrophages demonstrate a predominant M2 phenotype. To examine the effects of pioglitazone, the number of M1, M2, and mixed M1/M2 macrophages were quantitated. As shown in Figure 3A, pioglitazone treatment resulted in a decrease in total macrophages, along with a decrease in M1 and mixed M1/M2 macrophages, with a relative increase in M2 macrophages.

Previous studies have noted decreased capillaries in adipose tissue of insulin resistant subjects along with an increase in larger blood vessels [2], [4]. To assess the effects of pioglitazone treatment on adipose vascularity, we counted capillaries and large vessels, as described in Methods. As shown in Figure 3B, pioglitazone treatment resulted in a significant increase in adipose tissue capillaries, and no change in larger vessels.

**Figure 4:**
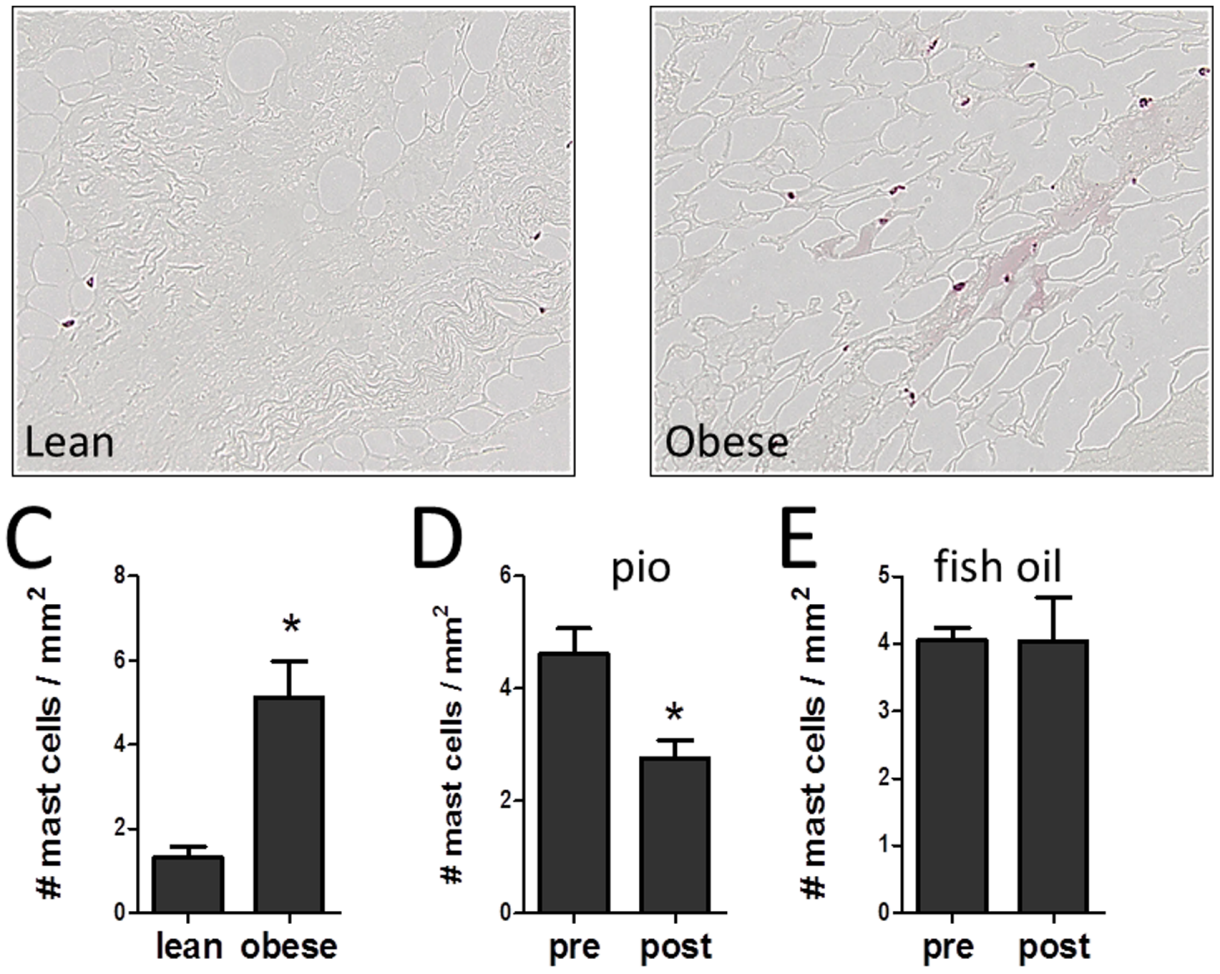
Quantitation of mast cells in adipose tissue. Mast cells were identified in adipose tissue by staining for tryptase in a representative (A) lean and (B) obese subject. C. Quantitation of mast cells in adipose tissue of lean and obese subjects. D. Mast cells in adipose tissue pre and post pioglitazone. E. Mast cells pre and post fish oil treatment.

Recent studies have noted the presence of mast cells in both rodent and human adipose tissue [15]–[17]. To identify mast cells, adipose tissue sections were stained with antibodies to tryptase, as described in methods, and the total number of mast cells in the whole section counted. As shown in Figure 4, mast cells were readily identifiable in the sections, and mast cells were counted. Mast cells can be found as isolated cells or in clusters. As shown in Figure S2, macrophages stained with CD68 and mast cells stained with tryptase are often found in the same region. Pioglitazone treatment reduced mast cells in adipose tissue by 35±9% (p<0.01, Figure 4D). The subjects treated with pioglitazone were all obese and had IGT, and therefore we wished to determine whether mast cells are present at a higher level in obese subjects. Therefore we compared mast cell number in a separate group of obese and lean subjects, whose characteristics are shown in Table 1. As shown in Figure 4C, the adipose tissue from obese subjects contained more mast cells than the lean subjects, and were comparable to the IGT subjects prior to pioglitazone treatment (Figure 4D). In recent studies, we treated obese subjects with ω-3 fatty acids and demonstrated a reduction in adipose tissue macrophages [12]. Therefore, we examined mast cell number in adipose tissue of subjects pre- and post-fish oil treatment. As shown in Figure 4E, there was no effect of fish oil treatment on mast cells. Hence, not all methods that result in improved adipose inflammation result in fewer mast cells.

## Discussion

Previous studies have characterized a number of pathological changes in adipose tissue in insulin resistant subjects, including an increase in inflammatory macrophages increased cytokine production, increased fibrosis and a more stiff ECM, and a decrease in capillaries [2]–[4], [6]. Weight loss, especially in response to bariatric surgery, is effective at improving adipose tissue inflammation [36]–[38], but relatively few drug treatments have been successful at improving the adipose tissue dysfunction.

A number of studies have examined the mechanism of action of TZDs, which improve peripheral insulin sensitivity [18], [39]. The treatment of either insulin resistant or diabetic humans with either pioglitazone or rosiglitazone reduces adipose inflammation and cytokine expression and increases the plasma level of anti-inflammatory adiponectin [40], [41]. Fish oils contain ω-3 fatty acids, which also have PPARγ agonist effects, and subjects treated with fish oil demonstrate improvements in adipose vascularity, inflammation and fibrosis, although no change in insulin sensitivity [12], [13], [42].

The mechanism of the improved insulin sensitivity of TZDs is likely multifactorial. On the one hand, TZDs have direct effects to induce apoptosis in macrophages [7]. In addition, TZD treatment results in an increase in subcutaneous adipose tissue and a reduction ectopic lipid in muscle and liver, and this relief of muscle and liver lipotoxicity would be expected to reduce insulin resistance at those sites [18], [43], [44]. A stiff ECM has been hypothesized to play a role in adipose dysfunction by causing worsened hypoxia, necrosis and inflammation with adipocyte expansion [5]. Therefore, it is important to understand the changes in adipose tissue following pioglitazone treatment, since this drug improves insulin sensitivity coincident with expanded adipose mass.

This study was designed to examine the mechanisms underlying pioglitazone and fish oil mediated changes in adipose tissue in insulin resistant subjects. As described above, a careful analysis of adipocyte size employed a new image analysis methodology that examined over 8,000 cells per histological section. Following pioglitazone treatment, there was an increase in overall fat cell size, with fewer small cells and more of the larger cells. Several previous studies have examined adipose cell size with TZD treatment, using different methods. Whereas some studies have shown an overall net increase in cell size, which would correspond with the increase in adipose mass [30], [31], other studies have found an increase in small adipocytes and a net decrease in fat cell size [32], [33], and another study found no change [34]. It is not clear why there is a discrepancy between studies. In addition to differences in patient population, there are methodological differences. The method described herein has the advantages of identifying cells histochemically in situ with a high level of accuracy without a collagenase digestion and of using an image analysis system that makes it feasible to size a large number of cells.

In spite of this overall increase in adipocyte size induced by pioglitazone, however, there was a reduction in macrophage number. Previous studies have demonstrated decreased macrophage number following pioglitazone treatment [2], [6], [45], but macrophage polarity has not been examined. In this study, there was a shift of macrophage polarity, with fewer pro-inflammatory M1 and relatively more M2 macrophages. Pioglitazone treatment decreases adipose inflammation and PPARγ agonists directly induced macrophage apoptosis [7], and it is possible that M1 macrophages are more sensitive to this apoptotic effect. Furthermore, PPARγ is required for alternative macrophage activation in mice, and PPARγ agonists increase mannose receptor expression in human monocytes, suggesting that PPARγ activation promotes the M2 phenotype [46]–[48]. It is important to recognize the complexity of macrophage phenotype [49], as illustrated by the fact that many adipose macrophages had a mixed M1/M2 staining.

Several recent studies have demonstrated the presence of mast cells in adipose tissue of both mice and humans [16], [17]. Mast cells have usually been described in relation to allergic disease and express histamine, prostaglandins, and proteases, but these cells also express a wide range of peptides related to inflammation, angiogenesis and fibrosis, all of which are involved in adipose pathology in insulin resistance [50]. In mice, the genetic depletion of mast cells led to resistance to the deleterious metabolic effects of a high fat diet [17]. In humans, mast cells tended to be found in the vicinity of blood vessels and fibrotic areas, correlated with other inflammatory markers, and were higher in diabetic humans [16], [17].

In this study, we found an increase in mast cell number with obesity, and also examined the effects of two different drugs that have demonstrated anti-inflammatory effects in adipose tissue. Mast cells were often found in areas with macrophages. Pioglitazone treatment not only reduced macrophage number and altered the polarity of the macrophages, but also reduced the number of mast cells in adipose tissue. ω-3 fatty acids have a broad spectrum of anti-inflammatory properties [11] along with PPARγ agonist effects [13], [14], [42]. In a recent study, we demonstrated a decrease in adipose tissue macrophages following 3 months of treatment of insulin resistant subjects with fish oils [12]. However, this decrease in macrophages from fish oils was not accompanied by a decrease in mast cells. It is possible that the mast cell reduction is directly dependent on the PPARγ effect, and fish oils are much less potent than pioglitazone as a PPARγ agonist; it is also possible that a longer duration of treatment or a higher dose of fish oil would have reduced mast cells in adipose.

In previous gene expression studies, insulin resistant subject demonstrated decreased VEGF mRNA and decreased capillary density, and also increased expression of components of the Fc-epsilon signaling pathway, which are involved in mast cell activation [51]. In contrast, the opposite effects occur to these genes in response to pioglitazone treatment, including increased VEGF, VEGF receptor, downstream components involved with angiogenesis, and decreased Fc-epsilon pathway components [24]. Although pioglitazone did not increase elastin gene expression, many proteases with the ability to degrade elastin fibers were down regulated [24]. In future studies, it would be useful to examine elastin degradation in the adipose of insulin resistant subjects in the context of capillary number and adipose inflammation.

These previous gene expression studies and the data described in this paper suggest possible mechanisms for the pioglitazone- mediated improvement in adipose tissue pathology in insulin resistance. In spite of larger adipocytes, pioglitazone increases expression of genes involved in capillary development, and also increases elastin. In a previous study, we examined elastin staining in adipose tissue and found less elastin in both fibrotic and non-fibrotic areas in obese insulin resistant subjects [4]. An increase in adipose tissue elastin would be predicted to allow the adipose tissue to be more flexible and better withstand the changes in adipose tissue architecture associated with adipocyte enlargement [5]. This would lead to less stress on the microvasculature, a higher number of capillaries, less hypoxia and together less adipocyte necrosis and inflammation.

## Supporting Information

Figure S1 **Bivariate comparison of manual measurements of cross-sectional area to automated measurement of cross-sectional area.**
(TIF)

Figure S2 (TIF)

Methods S1 (DOCX)
