# Supplementary figures and images for: Pioglitazone Treatment Reduces Adipose Tissue Inflammation through Reduction of Mast Cell and Macrophage Number and by Improving Vascularity
Source: PLoS One. 2014 Jul 10;9(7):e102190. doi: 10.1371/journal.pone.0102190 (PMC4092104; doi:10.1371/journal.pone.0102190)

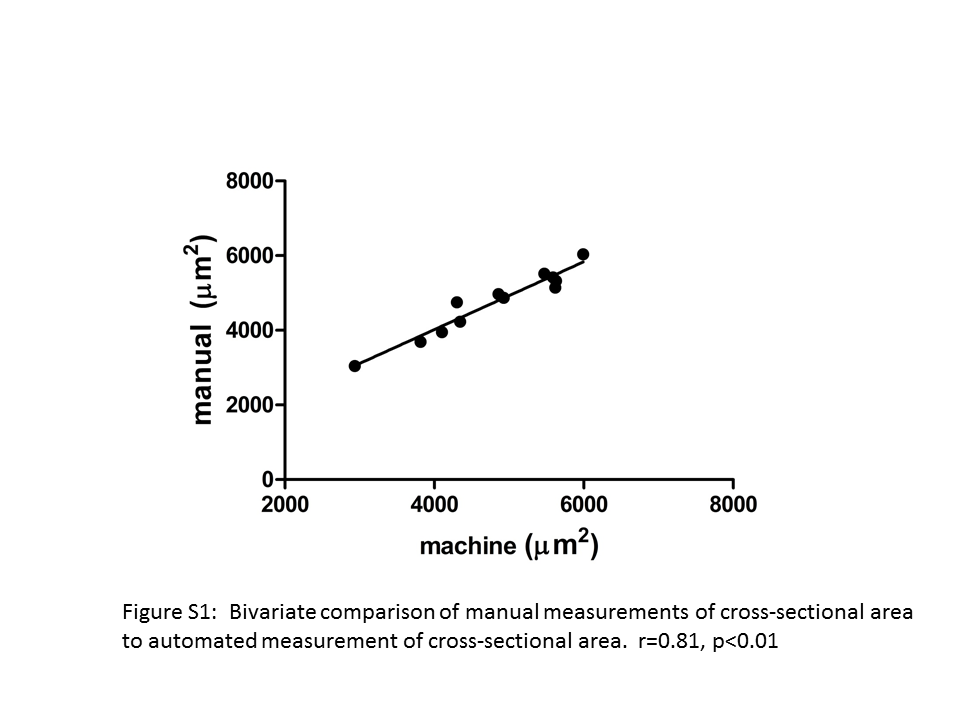

Supplement: Figure S1 — Bivariate comparison of manual measurements of cross-sectional area to automated measurement of cross-sectional area. (TIF) [file pone.0102190.s001.tif]

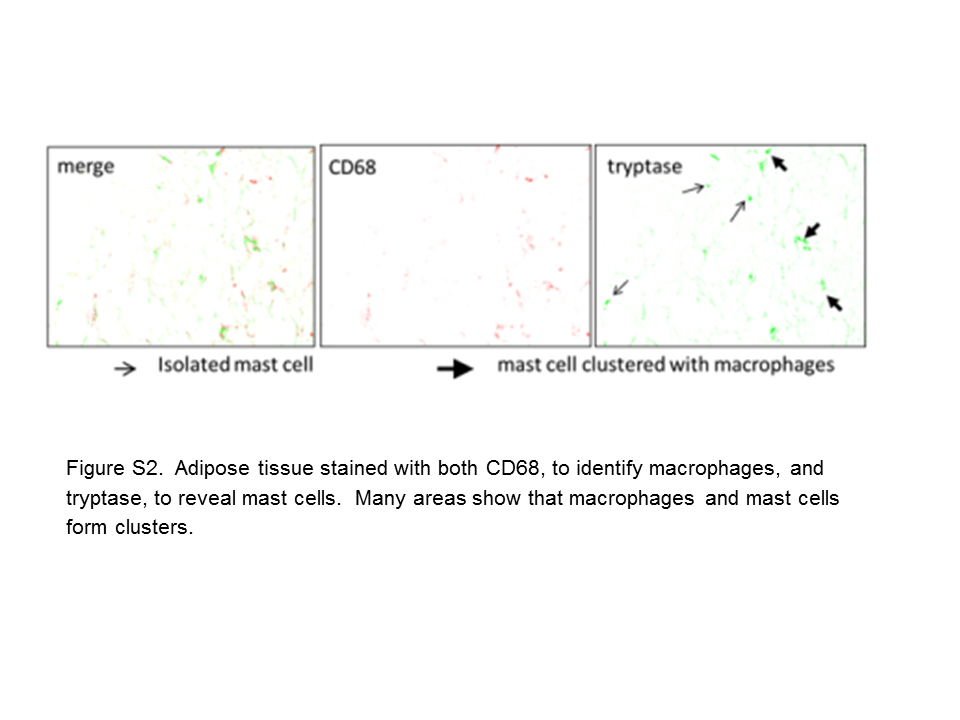

Supplement: Figure S2 — (TIF) [file pone.0102190.s002.tif]
