# Supplementary material for: Pioglitazone Treatment Reduces Adipose Tissue Inflammation through Reduction of Mast Cell and Macrophage Number and by Improving Vascularity
Source: PLoS One. 2014 Jul 10;9(7):e102190. doi: 10.1371/journal.pone.0102190 (PMC4092104; doi:10.1371/journal.pone.0102190)
Supplement: Methods S1 — (DOCX) [file pone.0102190.s003.docx]

**Supplementary Methods**

In this supplementary methods section, we will report a completely automated algorithm for adipocyte detection that was used for the cross-sectional area analysis (CSA). The automated image segmentation algorithm for adipocyte tissue contains two steps: 1) Machine learning based automatic detection of the geometric center of the adipocytes, and 2) a gradient vector field (GVF) snake based automatic segmentation that adopts color gradients.

**Machine learning based automated adipocyte detection:**

In this step, image textures (intensity distribution patterns) are used to as image features to distinguish adipocyte centers and boundaries. Textons [[1](#_ENREF_1)] are defined as repetitive local features that humans perceive as being discriminative between textures. We use the multiple scale Schmid filter bank [[2](#_ENREF_2)] composed of 13 rotation invariant filters:

$$F\left( r, \delta, \tau\right)=F_{0}\left( \delta,\tau\right)+\cos(\frac{\pi\gamma\tau}{\delta})e^{-\frac{r^{2}}{2\delta^{2}}}$$

The image filtering responses are clustered using *K-*means to generate a large codebook. A texton library is constructed from the corresponding cluster centers. The pixel-wise segmentation of imaged adipocytes is performed by automatic classification. Based on the labeled ground truth masks, 2000 positive and negative pixels are extracted from the adipocyte’s boundaries and non-boundary regions in the image. The appearance of the neighbors of each training pixel is modeled by a compact quantized description - texton histogram, where each pixel is assigned to its closest texton using the following equation:

$$h\left( i \right)=\sum_{j} count(T\left( j \right)=i),$$

where $I$ denotes the digitized adipocyte image, $i$is $i$-th element of the texton dictionary, and $T\left( j \right)$ returns the texton assigned to pixel $j$. The windowed texton histogram is computed around each individual image pixel.

After normalization, the texton histogram actually represents the texton channel frequency distribution in a local neighborhood around the centered pixel. In order to compensate for scale changes, the texton histogram is extracted from 3 different window sizes (4, 8, 16 pixels, respectively) and concatenated into one large feature vector. This concatenated texton histogram is used as image features to train the classifiers. An integral histogram [[3](#_ENREF_3)] is used to calculate the windowed texton histogram. The algorithm starts by exploiting the spatial arrangement of data points. It then recursively propagates an aggregated histogram. The aggregated histogram starts from the origin and traverses through the remaining points along a scan-line. At each step, a single bin is updated using the values of the integral histogram at the previous visited neighboring data points. The integral histogram method speeds up the automatic adipocyte image feature extraction significantly.

The Adaboost is chosen as the classifier for automatic segmentation. AdaBoost works by sequentially applying a classification algorithm on a reweighed version of the training data and produces a sequence of weak classifiers. The weak leaner used in our experiments is classification stump. The strong classifier is assembled from all the weak classifiers to minimize the cost function representing the classification accuracy. Given a test image, we apply the trained strong classifiers for each pixel and separate the image into adipocyte and non-adipocyte regions. Using the multiscale texton histogram, integral histogram, and AdaBoost, a fast and accurate pixel-wise segmentation algorithm can be implemented for detecting the geometric centers of the adipocytes.

**Deformable model based boundary delineation using color gradient**

The gradient vector field (GVF) deformable model algorithm was used to delineate the final adipocyte cell boundaries. The initial contours are driven to adipocyte boundaries by external force defined as:

$\nabla E_{ext}\left( X\left( s \right) \right)= -\Theta$,

where $X\left( s \right)=(x\left( s \right),y(s))$ with $x\left( s \right)$ and $y(s)$ denoting coordinates along the contour, $\Theta$ represents the GVF field defined as $\Theta\left( x, y \right)=\left[ u\left( x,y \right),v\left( x,y \right) \right]$.

The color image is first converted into grayscale image to compute $\Theta$. In gray-level images, the gradient is defined as the first derivative of the image luminance. It has a high value in those regions exhibiting high luminance contrast. However, simply transforming color images into gray-level images by taking the average of three RGB channels and applying the gray-level image gradient operator does not provide satisfactory results in our applications.

We adopt the definition of gradients for color images [[4-6](#_ENREF_4)] that can fully utilize the color information of digitized adipocyte images. In contrast to previous approaches, we define the color gradient in Luv color space rather than RGB color space, because Euclidean metrics and distances are perceptually uniform in Luv color space, which is not the case in RGB color space [[5](#_ENREF_5)].

Let ℾ$\left( x,y \right): \mathbb{R}^{3}$ be a color image, based on classical Riemannian geometry results. The norm can be written in matrix form:

$dℾ^{2}= \left[ \begin{aligned} dx \\ dy \end{aligned} \right]^{T}\left[ \begin{matrix} g_{11} & g_{12} \\ g_{21} & g_{22} \end{matrix} \right]\left[ \begin{aligned} dx \\ dy \end{aligned} \right]$,

where $g_{11}=[\partialℾ/\partial x]^{2}$, $g_{12}=g_{21}=(\partialℾ/\partial x)(\partialℾ/\partial y)$, $g_{22}=[\partialℾ/\partial y]^{2}$.

The quadratic form (8) achieves its extreme changing rates in the directions of the eigenvectors of matrix $\left[ g_{i,j} \right], i=1,2, j=1,2,$ and the changing magnitude is decided by its eigenvalues $\lambda_{+}$ and $\lambda_{-}$. In our approach, we select $\sqrt{\lambda_{+}-\lambda_{-}}$ to define the color gradient,

$\Theta= \sqrt{\lambda_{+}-\lambda_{-}}$,

where

$$g_{11}=\left[ \begin{aligned} |{\frac{\partial L}{\partial x}|}^{2} \\ {\frac{\partial u}{\partial x}|}^{2} \\ {\frac{\partial v}{\partial x}|}^{2} \end{aligned} \right] g_{22}=\left[ \begin{aligned} |{\frac{\partial L}{\partial y}|}^{2} \\ {\frac{\partial u}{\partial y}|}^{2} \\ {\frac{\partial v}{\partial y}|}^{2} \end{aligned} \right] g_{12}= g_{21}=\left[ \begin{aligned} |\frac{\partial^{2}L}{\partial x\partial y}| \\ |\frac{\partial^{2}u}{\partial x\partial y}| \\ |\frac{\partial^{2}v}{\partial x\partial y}| \end{aligned} \right]$$

where $L,u,v$ correspond to the three channels in Luv color space. The automatic calculation of the percentage of collagen and elastin is based on an adaptive thresholding followed by a connect component analysis to estimate the area of collagen and elastin.

**Reference**

1. Julesz, B., *Spatial nonlinearities in the instantaneous perception of textures with identical power spectra.* Philosophical Transactions of the Royal Society of London. B, Biological Sciences, 1980. **290**(1038): p. 83-94.

2. Schmid, C. *Constructing models for content-based image retrieval*. in *Computer Vision and Pattern Recognition, 2001. CVPR 2001. Proceedings of the 2001 IEEE Computer Society Conference on*. 2001. IEEE.

3. Porikli, F. *Integral histogram: A fast way to extract histograms in cartesian spaces*. in *Computer Vision and Pattern Recognition, 2005. CVPR 2005. IEEE Computer Society Conference on*. 2005. IEEE.

4. Di Zenzo, S., *A note on the gradient of a multi-image.* Computer Vision, Graphics, and Image Processing, 1986. **33**(1): p. 116-125.

5. Sapiro, G. and D.L. Ringach, *Anisotropic diffusion of multivalued images with applications to color filtering.* Image Processing, IEEE Transactions on, 1996. **5**(11): p. 1582-1586.

6. Gevers, T., *Adaptive image segmentation by combining photometric invariant region and edge information.* Pattern Analysis and Machine Intelligence, IEEE Transactions on, 2002. **24**(6): p. 848-852.
